# Evidence for zoonotic origins of Middle East respiratory syndrome coronavirus

**DOI:** 10.1099/jgv.0.000342

**Published:** 2016-02

**Authors:** Hui-Ju Han, Hao Yu, Xue-Jie Yu

**Affiliations:** ^1^​ School of Public Health, Shandong University, Jinan 250012, Shandong Province, PR China; ^2^​ School of Medicine, Fudan University, Shanghai 200433, PR China; ^3^​ Departments of Pathology and Microbiology and Immunology, University of Texas Medical Branch, Galveston, TX 77555-0609, USA; ^4^​ Center for Biodefense and Emerging Infectious Diseases, University of Texas Medical Branch, Galveston, TX 77555-0609, USA

## Abstract

Middle East respiratory syndrome (MERS) is an emerging infectious disease, caused by Middle East respiratory syndrome coronavirus (MERS-CoV) and is considered to be a zoonosis. However, the natural reservoirs of MERS-CoV remain obscure, with bats and camels as the most suspected sources. In this article, we review the evidence supporting a bat/camel origin of human MERS-CoV infection and current knowledge on the modes of camel-to-human transmission of MERS-CoV.

## Human coronaviruses (HCoVs)

Coronaviruses (CoVs) are enveloped, positive-sense RNA viruses with large genomes (29–32 kb) packaged in particles with corona-like morphology (Lai *et al.*, 2007). CoVs taxonomically belong to the subfamily *Coronavirinae*, family *Coronaviridae*, in the order *Nidoviales*, and can be further classified into four genera: *Alphacoronavirus*, *Betacoronavirus*, *Gammacoronavirus* and *Deltacoronavirus*, with the genus *Betacoronavirus* further divided into four genetic lineages, termed A–D (de Groot, 2011).

Six HCoVs have been identified to date: HCoV-229E, OC43, NL63, HKU1, severe acute respiratory syndrome (SARS) coronavirus (SARS-CoV) and Middle East respiratory syndrome (MERS) coronavirus (MERS-CoV). HCoV-229E and NL63 belong to the genus *Alphacoronavirus*, while the other four HCoVs belong to the genus *Betacoronavirus*, with HCoV-OC43 and HKU1 in lineage A, SARS-CoV in lineage B and MERS-CoV in lineage C (Zaki *et al.*, 2012).

SARS-CoV is the causative agent of SARS, which emerged as a previously unknown severe human respiratory infection in the Guangdong Province of China in 2002 (Drosten *et al.*, 2003; Ksiazek *et al.*, 2003). SARS is highly contagious, and by the end of the 2003 epidemic a total of 8098 probable SARS cases had been reported to the World Health Organization (WHO) from 29 countries, with 774 SARS-related deaths (case fatality rate: 9.6 %) (WHO, 2004). Before the discovery of SARS-CoV in 2003, only two HCoVs, namely HCoV-229E and OC43, were known (Lu *et al.*, 2012); however, these did not attract much attention as they only caused mild upper respiratory tract infections in humans (Wevers & van der Hoek, 2009). With surging interest in CoVs after the emergence of SARS, two additional novel HCoVs, NL63 and HKU1, were identified in 2004 and 2005, respectively (van der Hoek *et al.*, 2004; Woo *et al.*, 2005), which also caused only mild upper respiratory tract infections(Wevers and van der Hoek, 2009). However, in 2012, MERS attracted more attention as an emerging infectious disease because it caused severe lower respiratory infection and renal failure, similar to the clinical manifestation of SARS. The responsible agent was identified as a novel HCoV named MERS-CoV (Zaki *et al.*, 2012).

## MERS

The index case of MERS was initially recognized in the Kingdom of Saudi Arabia in June 2012, when an elderly Saudi Arabian man was admitted to a local hospital with acute pneumonia and later died of progressive severe respiratory illness and renal failure. A novel CoV (named MERS-CoV) was isolated from the sputum of the patient and was identified as the causative agent (Zaki *et al.*, 2012). Since then, MERS has been an epidemic in the Middle East, and travel-associated cases have been reported in a number of countries outside the Middle East. As of 7 July 2015, 1368 laboratory-confirmed cases of MERS, including at least 487 deaths, have been reported to WHO, and 26 countries have been affected since 2012, including countries from the Middle East, Africa, Europe, Asia and North America. The majority of cases (approx. 75 %) are reported from the Middle East (WHO, 2015).

## Evidence for bats as natural reservoirs of MERS-CoV

Bats have been implicated as the main reservoir of members of the genera *Alphacoronavirus* and *Betacoronavirus* (Li *et al.*, 2005; Woo *et al.*, 2012) and play pivotal roles in interspecies transmission of CoVs. This is best exemplified by SARS-CoV, which was shown to originate from Chinese horseshoe bats (Ge *et al.*, 2013; Lau *et al.*, 2005; Li *et al.*, 2005), and was probably transmitted directly to humans (Ge *et al.*, 2013) or through an intermediate host, such as the palm civet (Guan *et al.*, 2003).

MERS-CoV, together with bat CoV-HKU4 and -HKU5, phylogenetically belongs to lineage C in the genus *Betacoronavirus* (van Boheemen *et al.*, 2012). Thus, it is suspected that the emerging MERS-CoV might also originate from bats. Recent studies have revealed diverse types of MERS-related CoVs in bats from Saudi Arabia (Memish *et al.*, 2013a), Africa (Ithete *et al.*, 2013), Europe (Annan *et al.*, 2013) and Asia (Yang *et al.*, 2014). Studies on the receptor usage of MERS-CoV have also revealed a bat origin of MERS-CoV. An initial study found that the receptor used by MERS-CoV was different from the one used by SARS-CoV. The receptor may be conserved, since MERS-CoV can replicate in both bat and human cells (Müller *et al.*, 2012). Later, dipeptidyl peptidase 4 (DPP4 or CD26) was identified as the cellular receptor for MERS-CoV (Raj *et al.*, 2013). Bat CoV-HKU4, which is closely related to MERS-CoV, can also use DPP4 as a receptor to initiate cellular entry, and this discovery further supports the hypothesis that bats might be the natural reservoirs of MERS-CoV (Wang *et al.*, 2014).

## Evidence supporting camels as direct sources of MERS-CoV

The close phylogenetic relationship of human MERS-CoV isolates with those obtained from bats initially suggested that MERS-CoV might have originated from bats. However, bats were unlikely to be the direct source of the MERS outbreak, since MERS cases were rarely found to have a history of contact with bats. Therefore, other animals were searched as direct sources of zoonotic transmission of MERS-CoV.

Anecdotal reports mentioned contact of MERS cases with camels and goats, suggesting that livestock might be an intermediate reservoir for MERS-CoV (Albarrak *et al.*, 2012; Buchholz *et al.*, 2013; Drosten *et al.*, 2013; Milne-Price *et al.*, 2014). A research team found that 100 % (50/50) of retired racing camels from Oman and 14 % (15/105) of camels from Spain had antibodies against MERS in their serum (Reusken *et al.*, 2013b). The high seroprevalence of MERS-CoV in camels indicated that camels may play a major role in the spread of MERS-CoV among Middle Eastern countries.

Serological and molecular findings also indicated that camels were direct sources of human infection. Several groups reported a high seroprevalence of MERS-CoV or a closely related virus in camels across the Arabian Peninsula and parts of eastern and northern Africa (Corman *et al.*, 2014b; Hemida *et al.*, 2013; Meyer *et al.*, 2014; Perera *et al.*, 2013; Reusken *et al.*, 2013a), while MERS-CoV antibodies were not found in other species of livestock/leisure animals, including cattle, goats, sheep, horses and chickens (Hemida *et al.*, 2013; Reusken *et al.*, 2013a, b). Consistent with serological evidence, recent studies also found that MERS-CoV carried by camels was genetically highly similar to that detected in humans. An investigation of an outbreak on a farm in Qatar found that sequences of MERS-CoV in the nasal swabs from three of 14 seropositive camels were similar to those of two human cases on the same farm (Haagmans *et al.*, 2014). Near-full-genome sequences of MERS-CoV were also identified from nasal swabs from camels in Saudi Arabia, Oman and Egypt, and these isolates were genetically highly similar to those isolated from humans (Chu *et al.*, 2014; Hemida *et al.*, 2014; Nowotny & Kolodziejek, 2014). A full-genome sequence of MERS-CoV was obtained from a camel in Qatar, which was genetically highly similar to the human isolates, and the camel-derived MERS-CoV could efficiently replicate in human cells, providing further evidence for the zoonotic potential of MERS-CoV from camels (Raj *et al.*, 2014).

A virus culture study found that MERS-CoV isolated from camels in Saudi Arabia and Egypt could replicate in *ex vivo* cultures of human respiratory tract tissues, as did a prototype human MERS-CoV. The tropism of both camel and human MERS-CoV isolates in the human respiratory cell cultures, including bronchial non-ciliated epithelial cells, alveolar type II pneumocytes and lung endothelial cells, was in accordance with the distribution of the MERS-CoV cellular receptor, DPP4. In addition, both human and camel MERS-CoV isolates were weak inducers of innate immune responses in a human cell line, further suggesting phenotypic similarities between camel and human MERS-CoV isolates. The similarity of tropism and replication competence of MERS-CoV isolates from human and camels in *ex vivo* cultures of human respiratory tract tissues suggested that exposure to zoonotic MERS-CoV could lead to human infection (Chan *et al.*, 2014).

In sporadic cases, although no direction of transmission was implied (i. e camel-to-human vs human-to-camel transmission), strong evidence for transmission of MERS-CoV between camels and humans was found. In an outbreak investigation, MERS-CoV was isolated from three camels and two humans on the same farm, and MERS-CoV isolates from the camels were genetically identical to those isolated from the humans (Haagmans *et al.*, 2014). Another recent study showed that camels for slaughter in Qatar, where two of the earliest MERS cases occurred, evidenced a high proportion of nasal MERS-CoV shedding (62/105), and sequence analysis showed the circulation of at least five different virus strains on these premises. This suggests that this place was a driver of MERS-CoV circulation and a high-risk area for human exposure (Farag *et al.*, 2015). The strongest evidence of camel-to-human transmission came from a human case infected with MERS-CoV after exposure to infected camels, which showed that the viral genome from one of the camels was almost identical to the sequence of the human-derived virus (Memish *et al.*, 2014). A subsequent study confirmed the transmission of MERS-CoV from camels to humans, but not vice versa, since serological data indicated that MERS-CoV was already circulating in the camels but not in the patient before the human infection occurred (Azhar *et al.*, 2014).

In spite of these seroprevalence results, viral sequence findings in camels and reports of camel exposure prior to experiencing MERS, the role of camels as direct sources of human infection has remained controversial, because many human MERS cases had no apparent contact with camels. In addition, several investigations of MERS-CoV seroprevalence in people in contact with camels yielded negative results (Aburizaiza *et al.*, 2014; Hemida *et al.*, 2015; Memish *et al.*, 2015). The controversy faded with a population-based seroepidemiological survey of MERS-CoV in Saudi Arabia, which provided evidence of the role of camels in the zoonotic transmission of MERS-CoV to humans. The survey showed that seroprevalence of MERS-CoV antibodies was significantly higher in individuals exposed to camels than in the general population, and it estimated that a large number of young men who had contact with camels were infected with MERS-CoV but did not show apparent symptoms, and that they might be responsible for the human MERS cases without previous camel exposure experience (Müller *et al.*, 2015). A recent study also suggested that exposure to camels was a risk factor for infection, as MERS-CoV-neutralizing antibodies were detected only in healthy persons who had daily occupational contact with camels, but not in persons without such contact (Reusken *et al.*, 2015).

A nationwide survey was carried out to determine the prevalence of MERS-CoV infection in camels throughout the Kingdom of Saudi Arabia by examining archived serum samples of camels, and the results indicated that MERS-CoV had been circulating in camels in the country since at least 1992, and could be classified phylogenetically into clades that were correlated with outbreaks of the disease among humans (Alagaili *et al.*, 2014). Another study also found a high proportion of MERS-CoV-neutralizing antibodies in archived serum samples of camels from eastern Africa as early as 1983 (153/189), suggesting long-term MERS-CoV circulation in camels and the presence of unrecognized primary human MERS cases in a wider geographical region beyond the Arabian Peninsula (Müller *et al.*, 2014).

In all the findings discussed above, camels are considered the direct source of zoonotic transmission of MERS-CoV to humans.

## Modes of camel-to-human transmission of MERS-CoV

MERS-CoV sequences have been detected more commonly in nasal swabs than in rectal specimens of camels (Alagaili *et al.*, 2014; Hemida *et al.*, 2014). Infection of camels in the laboratory also confirmed susceptibility, with a large quantity of virus shedding from the upper respiratory tract (Adney *et al.*, 2014). Therefore, droplet transmission or direct contact with infected camels may be the most likely mode of camel-to-human transmission of MERS-CoV.

Direct contact with camels can only explain some of the primary cases, since some MERS cases did not report any direct contact with camels. Other possible routes for camel-to-human transmission include food-borne transmission through consumption of unpasteurized camel milk/raw meat and the medicinal use of camel urine. Camels are an important source of milk in some Middle East countries and parts of Africa, and more than half of the camel milk is sold as unpasteurized fresh or fermented milk to local and urban consumers in Saudi Arabia (Faye *et al.*, 2014). A survey found the presence of MERS-CoV RNA in the milk of camels actively shedding the virus (Reusken *et al.*, 2014a). Whether MERS-CoV in milk is secreted by infected camels or is introduced as a contaminant during the milking process needs further investigation. An experimental study of the stability of MERS-CoV in milk showed that viable viruses could still be recovered after 48 h regardless of reduction in virus titre, indicating that infection could happen by consumption of unpasteurized fresh raw milk (van Doremalen *et al.*, 2014). Consumption of undercooked meat from infected camels and handling of infected raw camel meat without proper protective equipment may also pose risks for getting MERS-CoV from camels. Camel urine is part of the traditional pharmacopoeia (Al-Yousef *et al.*, 2012), and is used as a natural remedy for a variety of ailments in Middle Eastern countries. These practices may represent risk factors for infection, and further studies are needed to prove their camel-to-human transmission potential.

An oral–faecal transmission mode was also suspected. Using protein intrinsic disorder prediction, MERS-CoV was placed into disorder group C and was likely to persist in the environment for a rather long period of time, and showed high oral–faecal transmission chances (Goh *et al.*, 2013).

A study of the stability of MERS-CoV under different environmental conditions found that MERS-CoV was more stable under low-temperature/low-humidity conditions, suggesting the potential for MERS-CoV to be transmitted via contact or fomite transmission due to prolonged environmental presence (van Doremalen *et al.*, 2013).

## Nosocomial transmission of MERS-CoV

Person-to-person transmission of MERS-CoV can occur among relatives in households and among patients and healthcare workers in healthcare settings (Assiri *et al.*, 2013; Memish *et al.*, 2013b). To date, the majority of secondary cases reported are nosocomial infections that have occurred in healthcare settings, largely affecting non-healthcare workers, as was seen in the dramatic increase of cases in Saudi Arabia from March 2014 and the recent 2015 MERS outbreak in the Republic of Korea (Lee & Wong, 2015b; Oboho *et al.*, 2015). The 2015 MERS outbreak in the Republic of Korea resulted in 186 human infections and 36 deaths (WHO, 2015), which was caused by nosocomial infection and was the largest MERS outbreak to date outside the Middle East. It presented the possibility of future outbreaks in other countries with exported MERS cases from the Middle East. With continuing spread of MERS-CoV to countries outside the Middle East and to all continents, MERS remains a severe public health problem, and possible consequences of further international spread could be serious in terms of the patterns of nosocomial transmission within healthcare settings.

In spite of its similar clinical features to SARS, MERS differs from SARS in the pattern of transmission. While SARS transmits rapidly among people, person-to-person transmission of MERS-CoV is mainly limited to households and hospitals. This may be due to the characteristic of MERS-CoV requiring close contact for transmission, or due to the fact that person-to-person transmission of MERS-CoV is controlled by early diagnosis and isolation of MERS patients and their close contacts, with the lessons learned from our past experience of dealing with emerging infectious diseases. Thus, MERS may become a pandemic disease if neglected.

## Prevention and control of MERS

In spite of frequent reports of nosocomial infection of MERS-CoV, human-to-human transmission is not sustainable and MERS is considered to be a zoonotic disease. To date, WHO has declared that the overall transmission patterns of MERS remain unchanged, i.e. multiple introductions from animals to humans and secondary transmission in healthcare settings (WHO, 2015). Therefore, identification of the zoonotic sources of MERS-CoV might guide control strategies at the human–animal interface to stop future human infection. If the spillover process of MERS-CoV from animals to humans could be stopped, we may be able to put an end to further nosocomial outbreaks in the Middle East and beyond.

Available serological studies have indicated that the seropositivity of MERS-CoV neutralizing antibodies is much lower in juvenile than in adult camels, suggesting that MERS-CoV infection in camels may target young animals (Meyer *et al.*, 2014; Müller *et al.*, 2014; Reusken *et al.*, 2013b, 2014b). In agreement with the serological findings, the detection rate of MERS-CoV RNA in the nasal and/or rectal swabs of juvenile camels was higher than in those of adult camels (Alagaili *et al.*, 2014). In addition, a recent study found that MERS-CoV mainly targeted camels of less than 4 years of age, particularly calves, and the infection in juvenile camels manifested as an acute, epidemic and time-limited infection. Thus, delaying the social separation of calves or avoiding contact with camels aged less than 4 years might be a simple but effective measure to reduce spillover of MERS-CoV from camels to humans (Wernery *et al.*, 2015).

Although there is no evidence of sustained human-to-human transmission of MERS-CoV, nosocomial infection may sometimes lead to MERS outbreaks. The recent MERS outbreak in the Republic of Korea, the largest MERS outbreak ever recorded outside of Saudi Arabia, was a result of nosocomial transmission: a single exported case with a travel history in the Middle East resulted in 185 laboratory-confirmed human infections in Korea and one in China, with 36 deaths (WHO, 2015). The outbreak pattern in Korea was similar to the hospital outbreaks that occurred in the Middle East, which were attributed to failures of infection prevention and control in healthcare settings (Lee & Wong, 2015; Oboho *et al.*, 2015). Therefore, early diagnosis, prompt isolation of suspected cases and timely contact tracing of case contacts are key strategies to prevent nosocomial transmission.

## Conclusion

MERS-CoV is phylogenetically closely related to CoVs isolated from bats and camels; however, direct bat-to-human transmission is unlikely. Current studies have demonstrated the potential role of camels as direct sources of human infection of MERS-CoV. Interestingly, a study hypothesized that camels may acquire MERS-CoV from bats in Africa, which were then transported to the Middle East, where they transmitted the virus to non-immune humans (Corman *et al.*, 2014a).

Although the evidence linking MERS-CoV transmission from camels to humans is irrefutable, it is still important to conduct investigations where MERS is endemic to evaluate further the role of other animal species in the transmission of MERS-CoV. Moreover, modes of camel-to-human transmission have not been fully elucidated. Our current understanding of camel-to-human transmission of MERS-CoV is that MERS-CoV entered the human population in the Arabian Peninsula on multiple occasions from direct or indirect contact with infected camels or camel-related products (e.g. raw camel milk, camel urine). Further efforts are required to prove the effectiveness of these modes of camel-to-human transmission.
